# Multiplexed CRISPR/Cas9 Editing of Tumor Suppressor Genes in the Mouse Endometrium Recapitulates High-Risk Endometrial Carcinoma

**DOI:** 10.34133/cancomm.0010

**Published:** 2026-02-06

**Authors:** Maria Vidal-Sabanés, Raúl Navaridas, Núria Bonifaci, Ada Gay-Rua, Damià Ortega-Peinado, Joaquim Egea, Mario Encinas, Xavier Matias-Guiu, David Llobet-Navas, Xavier Dolcet

**Affiliations:** ^1^ Developmental and Oncogenic Signaling Group, Department of Basic Medical Sciences, University of Lleida, Biomedical Research Institute of Lleida (IRBLleida), Lleida 25198, Lleida, Spain.; ^2^ Herbert Irving Comprehensive Cancer Center, Vagelos College of Physicians and Surgeons, Columbia University Irving Medical Center, New York, NY 10032, USA.; ^3^ Molecular Mechanisms and Experimental Therapy in Oncology–Oncobell Program, Bellvitge Biomedical Research Institute (IDIBELL), L’Hospitalet de Llobregat, 08907 Barcelona, Spain.; ^4^ Biomedical Research Networking Center in Oncology (CIBERONC), Carlos III Health Institute (ISCIII), 28029 Madrid, Spain.; ^5^ Oncologic Pathology Group, Department of Basic Medical Sciences, University of Lleida, Biomedical Research Institute of Lleida (IRBLleida), Lleida 25198, Lleida, Spain.

Endometrial carcinoma (EC) is the fourth most common cancer in women in the Western world and the most frequent malignancy of the female genital tract, with an incidence of 10 to 20 per 100,000 women annually [1]. Traditional classifications distinguish Type I endometrioid EC from Type II non-endometrioid EC [2], while histological schemes categorize tumors into endometrioid endometrial carcinoma, clear cell carcinoma (CCC), serous endometrial carcinoma (SEC), and uterine carcinosarcoma (UCS). More recently, the Cancer Genome Atlas proposed a molecular classification defining ultramutated with polymerase ε mutations, hypermutated with microsatellite instability, copy-number low, and copy-number high (CNH) serous-like subtypes [3]. High-risk tumors, particularly UCS and SEC, are strongly associated with tumor protein 53 (*TP53*) mutations, extensive genomic alterations, poor prognosis, and disproportionately high mortality rates [4]. Despite these insights, the contribution of individual and combined tumor suppressor gene (TSG) mutations to EC progression remains incompletely understood, and faithful animal models of aggressive EC subtypes are scarce.

To address this, we applied an in vivo multiplex clustered regularly interspaced short palindromic repeats (CRISPR)/Cas9 strategy [5] targeting the 10 most frequently mutated TSGs in high-risk EC. Detailed materials and methods are provided in the Supplementary Materials. For gene selection, we analyzed genomic data from 1,133 EC cases across 8 studies in cBioPortal. After filtering for mutation profiles, histological grade, and survival data, we identified 334 high-risk cases (grade 3 endometrioid carcinoma, SEC, CCC, and UCS) and 479 low-risk endometrioid cases (grades 1 and 2). Comparative analysis confirmed poorer survival in the high-risk cohort and guided the selection of 10 recurrently mutated TSGs for functional modeling: *TP53*, protein phosphatase 2 scaffold subunit alpha (*PPP2R1A*), F-box and WD repeat domain containing 7 (*FBXW7*), chromodomain helicase DNA binding protein 4 (*CHD4*), rho GTPase activating protein 35 (*ARHGAP35*), phosphoinositide-3-kinase regulatory subunit 1 (*PIK3R1*), phosphatase and tensin homolog (*PTEN*), AT-rich interactive domain-containing protein 1a (*ARID1A*), mucin 16 (*MUC16*), and lysine methyltransferase 2D (*KMT2D*) (Fig. S1A).

Cas9-ribonucleoproteins (RNPs) targeting each locus were validated in vitro using synthetic DNA substrates, confirming the efficient cleavage of all targets (Fig. S1B and C). In vivo validation in mTmG reporter mice demonstrated editing capacity, as loxP-targeting RNPs induced green fluorescent protein (GFP) expression in electroporated uterine epithelium (Fig. S1D to G). Amplicon sequencing confirmed edits at all targeted genes, though at low aggregate frequencies, consistent with the small proportion of transfected epithelial cells (Fig. S1H). Histological analysis of uteri electroporated with no RNP, Cas9 alone, or sgRNA alone showed no morphological abnormalities for up to 16 weeks after intervention (Fig. S1I). Importantly, the edited endometrial epithelial cells remained viable and could be expanded in culture, enabling the quantification of editing efficiency and assessment of functional consequences (Fig. S1J to L).

By pooling Cas9-RNPs for intrauterine electroporation, we aimed to recapitulate the genetic heterogeneity underlying high-risk EC [6]. To assess heterogeneity at single-cell resolution, we adapted multiplexed rolling circle amplification (RCA) using padlock probes targeting wild-type transcripts of the 10 genes. Loss of detectable wild-type signal indicated CRISPR editing (Fig. S2A and B). Control experiments confirmed the specificity and multiplexing capability of the system, including the successful detection of *PTEN* loss in knockout mice (Fig. S2C and D). RCA applied to wild-type endometrial cells (Fig. S2E to K) or cells electroporated with pooled RNPs revealed striking cell-to-cell variability: while some cells retained transcripts for all genes, others showed loss of multiple TSG mRNAs (Fig. 1A to C and Fig. S2L and M). These results confirm that multiplexed editing generates a genetically mosaic tissue, more closely resembling human tumorigenesis than uniform conditional knockouts.

**Fig. 1.**
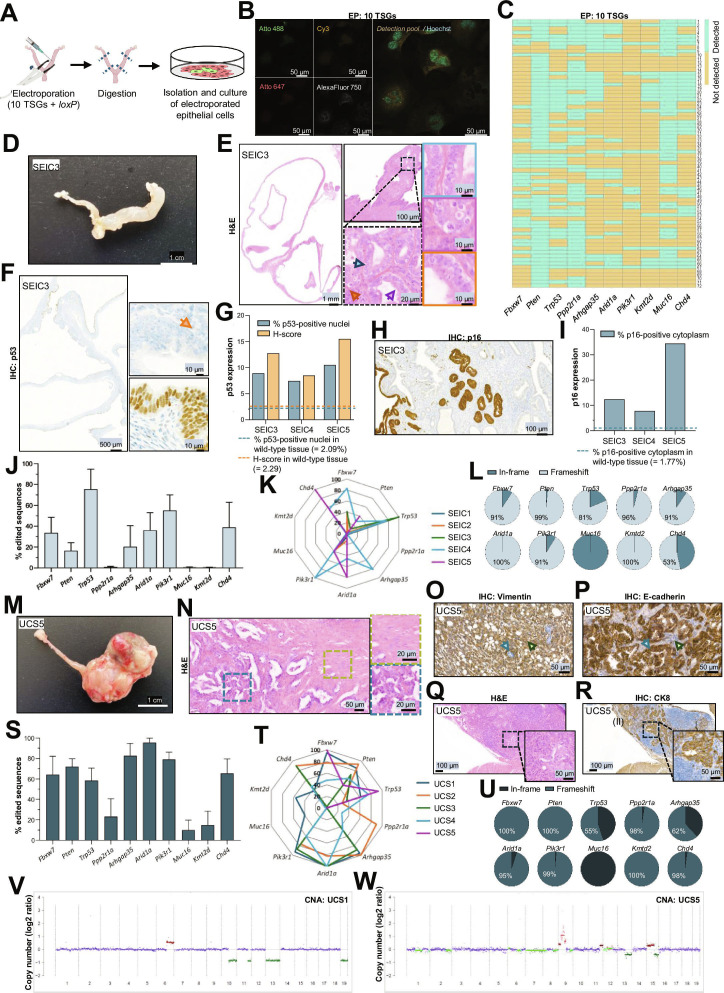
Intrauterine deletion of tumor suppressor genes leads to the formation of serous endometrial intraepithelial carcinomas and uterine carcinosarcomas with morphologic and molecular features of human pathology. (A) Workflow for electroporated endometrial epithelial cells culture. (B) Representative image of pooled detection of selected TSGs in electroporated with a pool of RNPs (EP: 10 TSGs), detected with fluorochrome code. Images show detection with Atto488 (green), Cy3 (orange), Atto647 (red), and/or AlexaFluor750 (gray). Hoechst (blue) is used for nuclear staining. (C) Quantification and colocalization of RNA spots according to the fluorochrome code at a single-cell resolution. Binary heatmap represents the presence (green) or absence (yellow) of *Fbxw7, Pten, Trp53, Ppp2r1a, Arhgap35, Arid1a, Pik3r1, Muc16, Kmt2d*, or *Chd4* detection in each cell (columns). (D) Macroscopic image from the uterus displaying SEIC (SEIC3). (E) H&E staining of case SEIC3, highlighting typical morphological characteristics in serous pathology. Blue arrows and squares show pleomorphic nuclei; purple arrows and squares show apoptotic cells, and orange arrows and squares show mitotic cells. (F) IHC images of p53 in case SEIC3, underlining aberrant p53 expression as a null pattern (upper amplification, orange arrow indicates p53 internal positive control) and overexpression pattern (lower amplification). (G) Quantification of p53-positive nuclei and p53 H-score in cases SEIC3, SEIC4, and SEIC5. Blue dashed line represents the percentage of p53-positive nuclei or p53 H-score in a wild-type endometrium. (H) IHC images of p16 expression in SEIC3. (I) Quantification of p16-positive cells in cases SEIC3, SEIC4, and SEIC5. Discontinuous horizontal lines represent the percentage of p16-positive cells in a wild-type endometrium. (J) Normalized frequency of edition of selected tumor suppressor genes (*Fbxw7*, *Pten*, *Trp53*, *Ppp2r1a*, *Arhgap35*, *Arid1a*, *Pik3r1*, *Muc16*, *Kmt2d*, and *Chd4*) from SEIC cases (SEIC1 to SEIC5). Data are presented as mean ± SEM. (K) Spider plot showing the normalized edition frequencies of targeted TSGs across SEIC samples (SEIC1 to SEIC5). Each axis represents one gene (*Fbxw7*, *Pten*, *Trp53*, *Ppp2r1a*, *Arhgap35*, *Arid1a*, *Pik3r1*, *Muc16*, *Kmt2d*, and *Chd4*) and the radial distance corresponds to the normalized edition frequency. (L) Pie charts showing the proportion of mutation type in edited genes from SEIC samples (SEIC1 to SEIC5). Data are presented as the mean of in-frame/frameshift mutations proportion from all samples. (M) Macroscopic image from uteri displaying UCS (case UCS5). (N) H&E staining of case UCS5. Squares in blue indicate the epithelial compartment, and squares in green show the mesenchymal compartment. (O and P) Representative IHC images of Vimentin (O) and E-cadherin (P) detections in case UCS5. Blue arrows indicate the epithelial compartment, and green arrows indicate the mesenchymal compartment. (Q) H&E of lymph node metastasis in case UCS5. (R) IHC of CK8 of lymph node metastasis in case UCS5. (S) Normalized frequency of edition of selected tumor suppressor genes (*Fbxw7, Pten, Trp53, Ppp2r1a, Arhgap35, Arid1a, Pik3r1, Muc16, Kmt2d*, and *Chd4*) from UCS cases (UCS1 to UCS5). Data are presented as mean ± SEM. (T) Spider plot showing the normalized edition frequencies of targeted TSGs across UCS samples (UCS1 to UCS5). Each axis represents one gene (*Fbxw7, Pten, Trp53, Ppp2r1a, Arhgap35, Arid1a, Pik3r1, Muc16, Kmt2d*, and *Chd4*) and the radial distance corresponds to the normalized edition frequency. (U) Pie charts showing the proportion of mutation type in edited genes from UCS samples (UCS1 to UCS5). Data are presented as the mean of in-frame/frameshift mutations proportion from all samples. (V and W) CNAs in UCS1 (V) and UCS5 (W). CNAs were estimated using the ichorCNA algorithm. The *X*-axis represents genomic coordinates concatenated by chromosome (1 to 22), and the *Y*-axis displays the log_2_ copy number ratio for each genomic bin. Each data point is color-coded according to the estimated integer copy number: 1 copy = green, 2 copies = blue, 3 copies = brown, ≥4 copies = red. Horizontal lines represent the median log_2_ ratio of each clonal CNA value and are colored according to copy-number state; subclonal CNAs are visible only as individual points without segment lines. Dark green points and segments indicate clonal single-copy losses, whereas light green points and segments mark subclonal single-copy losses. These profiles reflect the expected resolution of shallow whole-genome sequencing and ichorCNA’s probabilistic segmentation model. Abbreviations: *Arhgap35*, rho GTPase activating protein 35; *Arid1a*, AT-rich interaction domain 1A; *Chd4*, chromodomain-helicase-DNA-binding protein 4; CK8, cytokeratin 8; CNA, copy number alterations; EP, electroporated; *Fbxw7*, F-box and WD repeat domain containing 7; H-score, histoscore; H&E, hematoxylin and eosin; IHC, immunohistochemistry; *Kmt2d*, lysine methyltransferase 2D; *Muc16*, mucin 16; *Pik3r1*, phosphoinositide-3-kinase regulatory subunit; *Ppp2r1a*, protein phosphatase 2 scaffold subunit alpha; *Pten*, phosphatase and tensin homolog; RNP, ribonucleoprotein; SEIC, serous endometrial intraepithelial carcinoma; *Trp53*, transformation-related protein 53; TSG, tumor suppressor gene; UCS, uterine carcinosarcoma.

We electroporated 14 mice with pooled TSG RNPs and monitored them longitudinally (Fig. S3A). Ten developed visible uterine lesions, while 4 remained macroscopically normal. Histopathology identified 2 lesion types: serous endometrial intraepithelial carcinoma (SEIC) and UCS (Fig. S3B to D). SEICs displayed classical serous features [7], including macroscopic enlargement (Fig. 1D and Fig. S4A), marked nuclear atypia, epithelial stratification, papillary growth, high mitotic activity, and apoptotic figures (Fig. 1E and Fig. S4B). Immunohistochemistry (IHC) revealed abnormal p53 staining patterns, with both null and strong nuclear phenotypes (Fig. 1F and G and Fig. S4C and D), and diffuse cytoplasmic p16 expression compared to wild-type endometrium (Fig. 1H and I and Fig. S4E and F), reflecting diagnostic characteristics of human SEIC [8]. Next-Generation Amplicon Sequencing (NGAS) confirmed indels in all targeted TSGs, with *TP53* edits being particularly enriched (Fig. 1J to L).

UCS lesions exhibited distinct macroscopic features and characteristic biphasic morphology with separate carcinomatous and sarcomatous components [9] (Fig. 1M and N and Fig. S5A and B). Immunostaining confirmed epithelial–mesenchymal transition: vimentin marked sarcomatous areas while E-cadherin and cytokeratin 8 (CK8) labeled epithelial regions (Fig. 1O and P and Fig. S5C to E). All UCS lesions invaded the myometrium, and notably, one case showed lymph node metastasis, confirmed by CK8 IHC (Fig. 1Q and R). NGAS revealed heterogeneous disruptions across all 10 TSGs (Fig. 1S to U). Given that UCS is often associated with CNH serous-like tumors, we evaluated genomic stability in 2 cases by shallow whole-genome sequencing. UCS1 exhibited a largely copy-number neutral profile, with a few focal deletions and gains, consistent with a genomically stable tumor (Fig. 1V and Fig. S5F). In contrast, UCS5 displayed extensive chromosomal instability, including high-level amplifications across multiple chromosomes and pronounced subclonality, despite near-diploid ploidy (Fig. 1W and Fig. S5F and G). Interestingly, UCS1 developed within 13 weeks, whereas UCS5 required nearly 1 year to progress, suggesting that prolonged evolution may promote the accumulation of chromosomal instability. These results recapitulate the heterogeneity seen in human UCS, where some tumors arise from copy number-low molecular subtypes, while others display highly unstable genomes.

Unexpectedly, some macroscopically and histologically normal uteri harbored oncogenic mutations (Fig. S6A and B). Electroporation efficacy was confirmed by GFP staining in mTmG mice, excluding failed transfection as a possible explanation (Fig. S6C). Amplicon sequencing revealed indels in up to 5 TSGs, including *TP53* and *PTEN*, most of which produced frameshift mutations (Fig. S6D and E). These findings demonstrate that acquisition of multiple oncogenic mutations alone is insufficient to induce malignant transformation, supporting a multistep model of EC evolution. This observation aligns with human studies identifying clonal driver mutations in histologically normal endometrium [10] and emphasizes the role of permissive cellular or epigenetic contexts in tumor initiation.

Together, our findings establish that multiplexed in vivo CRISPR/Cas9 editing of high-risk TSGs faithfully reproduces the histological and molecular diversity of aggressive EC. Notably, all observed lesions were SEIC or UCS, recapitulating human high-risk EC subtypes, while no low-grade endometrioid carcinomas emerged. This highlights the specificity of the targeted TSGs in driving aggressive histology. The system’s ability to generate mosaic populations of mutant and wild-type cells within the same tissue more closely mirrors the clonal architecture of human tumors and provides a powerful tool for dissecting cooperative mutational interactions that enable clonal selection and drive tumor expansion.

Although multiple strategies exist for delivering CRISPR/Cas9, including viral vectors, lipid nanoparticles, extracellular vesicles, and physical transfection, our approach offers distinct advantages (Table S1) and provides a preclinical framework for testing these therapeutic strategies. Tumors generated through multiplex editing displayed heterogeneity in histology, mutational spectrum, and genomic stability, modeling the diversity observed clinically. The presence of oncogenic mutations in histologically normal tissue raises important questions regarding early detection and prevention, as it suggests a latent reservoir of genetically altered cells that may progress only under specific contexts.

In conclusion, multiplex in vivo CRISPR/Cas9 editing of 10 recurrently mutated TSGs faithfully recapitulated the molecular, morphological, and genomic hallmarks of high-risk EC, generating SEIC and UCS lesions. The model captures clonal heterogeneity, reveals oncogenic mutations in normal tissues, and reproduces chromosomal instability in human UCS. By linking genomic data to functional validation, it offers a rapid and versatile platform for mechanistic studies, therapeutic testing, and deeper insights into tumor evolution in aggressive gynecologic cancers.

## Ethical Approval

All procedures performed in this study followed the National Institute of Health Guide for the Care and Use of Laboratory Animals and were compliant with the guidelines of the Universitat de Lleida (permit number: N. 02-02/19).

## Data Availability

The data analyzed during the current study are available from the corresponding author upon reasonable request. The remaining data are available within the article or the Supplementary Materials.

## References

[1] Makker V, MacKay H, Ray-Coquard I, Levine DA, Westin SN, Aoki D, Oaknin A. Endometrial cancer. Nat Rev Dis Primer. 2021;7(1):88. 10.1038/s41572-021-00324-8 PMC9421940 34887451

[2] Bokhman JV. Two pathogenetic types of endometrial carcinoma. Gynecol Oncol. 1983;15(1):10–17. 6822361 10.1016/0090-8258(83)90111-7

[3] Cancer Genome Atlas Research Network, Kandoth C, Schultz N, Cherniack AD, Akbani R, Liu Y, Shen H, Robertson AG, Pashtan I, Shen R, et al. Integrated genomic characterization of endometrial carcinoma. Nature. 2013;497(7447):67–73. 23636398 10.1038/nature12113 PMC3704730

[4] Bell DW, Ellenson LH. Molecular genetics of endometrial carcinoma. Annu Rev Pathol. 2019;14:339–367. 30332563 10.1146/annurev-pathol-020117-043609

[5] Navaridas R, Vidal-Sabanés M, Ruiz-Mitjana A, Perramon-Güell A, Megino-Luque C, Llobet-Navas D, Matias-Guiu X, Egea J, Encinas M, Bardia L, et al. Transient and DNA-free in vivo CRISPR/Cas9 genome editing for flexible modeling of endometrial carcinogenesis. Cancer Commun. 2023;43(5):620–624. 10.1002/cac2.12409 PMC10174088 36762520

[6] Navaridas R, Vidal-Sabanés M, Ruiz-Mitjana A, Altés G, Perramon-Güell A, Yeramian A, Egea J, Encinas M, Gatius S, Matias-Guiu X, et al. In vivo intra-uterine delivery of TAT-fused Cre recombinase and CRISPR/Cas9 editing system in mice unveil histopathology of Pten/p53-deficient endometrial cancers. Adv Sci Weinh Baden-Wurtt Ger. 2023;10(32): Article e2303134. 10.1002/advs.202303134 PMC10646277 37749866

[7] Bogani G, Ray-Coquard I, Concin N, Ngoi NYL, Morice P, Enomoto T, Takehara K, Denys H, Nout RA, Lorusso D, et al. Uterine serous carcinoma. Gynecol Oncol. 2021;162(1):226–234. 33934848 10.1016/j.ygyno.2021.04.029 PMC9445918

[8] Gatius S, Matias-Guiu X. Practical issues in the diagnosis of serous carcinoma of the endometrium. Mod Pathol. 2016;29:S45–S58. 26715173 10.1038/modpathol.2015.141

[9] Bogani G, Ray-Coquard I, Concin N, Ngoi NYL, Morice P, Caruso G, Enomoto T, Takehara K, Denys H, Lorusso D, et al. Endometrial carcinosarcoma. Int J Gynecol Cancer. 2023;33(2):147–174. 36585027 10.1136/ijgc-2022-004073

[10] Lee-Six H, Olafsson S, Ellis P, Osborne RJ, Sanders MA, Moore L, Georgakopoulos N, Torrente F, Noorani A, Goddard M, et al. The landscape of somatic mutation in normal colorectal epithelial cells. Nature. 2019;574(7779):532–537. 31645730 10.1038/s41586-019-1672-7

