# Supplementary material for: Multiplexed CRISPR/Cas9 Editing of Tumor Suppressor Genes in the Mouse Endometrium Recapitulates High-Risk Endometrial Carcinoma
Source: Cancer Commun (Lond). 2026 Feb 6;46:0010. doi: 10.34133/cancomm.0010 (PMC12876560; doi:10.34133/cancomm.0010)
Supplement: Supplementary 1 — Supplementary Methods Tables S1 to S11 Figs. S1 to S6 [file cancomm.0010.f1.docx]

**SUPPLEMENTARY MATERIALS**

**Multiplexed CRISPR/Cas9 editing of tumor suppressor genes in the mouse endometrium recapitulates high-risk endometrial carcinoma**

MARIA VIDAL-SABANÉS^1^, RAÚL NAVARIDAS^2^, NÚRIA BONIFACI^3,4^, ADA GAY-RUA^1^, DAMIÀ ORTEGA-PEINADO^1^, JOAQUIM EGEA^1^, MARIO ENCINAS^1^, XAVIER MATIAS-GUIU^4,5^, DAVID LLOBET-NAVAS^3,4^, XAVIER DOLCET^1,4,*^

**Affiliations**

1. Developmental and Oncogenic Signaling Group, Department of Basic Medical Sciences, University of Lleida, Biomedical Research Institute of Lleida (IRBLleida), Lleida 25198, Lleida, Spain.
2. Herbert Irving Comprehensive Cancer Center, Vagelos College of Physicians and Surgeons, Columbia University Irving Medical Center, New York 10032 NY, United States of America.
3. Molecular Mechanisms and Experimental Therapy in Oncology–Oncobell Program, Bellvitge Biomedical Research Institute (IDIBELL), L'Hospitalet de Llobregat 08907, Barcelona, Spain.
4. Biomedical Research Networking Center in Oncology (CIBERONC), Carlos III Health Institute (ISCIII), Madrid 28029, Madrid, Spain.
5. Oncologic Pathology Group, Department of Basic Medical Sciences, University of Lleida, Biomedical Research Institute of Lleida (IRBLleida), Lleida 25198, Lleida, Spain.

**^*^Correspondence author**

**Xavier Dolcet, Ph.D.** Institut de Recerca Biomèdica de Lleida (IRBLleida)

Email: xavi.dolcet@udl.cat

**Supplementary Methods**

**1. Experimental mouse models**

Mice were housed in a barrier facility, and pathogen-free procedures were used in all mouse rooms. Animals were under 12 hours of light/dark cycles at 22°C, and they had *ad libitum* access to water and food. All procedures were performed according to the guidelines of the Ethical Committee of Universitat de Lleida and the National Institute of Health Guide for the Care and Use of Laboratory Animals. Homozygous membrane-targeted tandem dimer Tomato/membrane-targeted Green Fluorescent Protein (mTmG) reporter (B6.129(Cg)-*Gt(ROSA)26Sor^tm4(ACTB-tdTomato,-EGFP)Luo^*/J) were obtained from the Jackson Laboratory (Bar Harbor, ME, USA). Wild-type C57BL/6J were bred in the Universitat de Lleida animal housing facility. Primers and PCR conditions for genotyping are detailed in **Supplementary Table S2**.

**2. Preparation of CRISPR/Cas9-ribonucleoprotein**

RNP preparation was performed as previously described [1] with some modifications. CRISPR RNAs (crRNAs) and trans-activating CRISPR RNAs (tracrRNA) were obtained from Integrated DNA Technologies (IDT). Sequences of crRNA used for this work are detailed in **Supplementary Table S3**. Lyophilized crRNAs and tracrRNA were resuspended at 100 μmol/L in nuclease-free duplex buffer, containing 100 mmol/L potassium acetate and 30 mmol/L 4-(2-hydroxyethyl)-1-piperazineethanesulfonic acid (HEPES), pH 7.5 (IDT, 11-05-01-03). For crRNA:tracrRNA hybridization, both were mixed at equimolar concentration and diluted in nuclease-free duplex buffer to a 20 μmol/L concentration. Then, RNAs were heated at 95°C for 5 minutes and subjected to a negative temperature gradient (-2°C/30 sec) in a thermal cycler until they reached room temperature. Hybridized 20 μmol/L crRNA:tracrRNA (single guide RNA, sgRNA) stocks were stored at -20 °C. Recombinant Streptococcus pyogenes Cas9 (Alt-R® S.p. Cas9 Nuclease V3, IDT, 1081059) was mixed at equimolar concentration with sgRNA at a final concentration of 15 μmol/L in 4 μL (0.5 μL of 61 μmol/L Cas9 and 1.5 μL of 20 μmol/L sgRNA). The mixture was incubated for 20 minutes at room temperature to allow Cas9-sgRNA RNP formation.

**3. *In vitro* CRISPR/Cas9 ribonucleoprotein assay**

To test the Cas9 nuclease on-target activity of *loxP*-RNP, *Fbxw7*-RNP, *Pten*-RNP, *Trp53*-RNP, *Ppp2r1a*-RNP, *Arhgap35*-RNP, *Arid1a*-RNP, *Pik3r1*-RNP, *Muc16*-RNP, *Kmt2d*-RNP, and *Chd4*-RNP, oligonucleotides containing target sequences in tandem were used (CRISPR tandem assays). Oligonucleotides were obtained lyophilized at IDT and were resuspended at a final concentration of 10 μmol/L in nuclease-free water. To obtain double-chain DNA fragments, oligonucleotides were amplified through a PCR reaction. Oligonucleotide sequences, as well as primers and PCR conditions are detailed in **Supplementary Table S4** and **Supplementary Table S5**, respectively. PCR amplicons were resolved in agarose gels and purified using the NucleoSpin Gel and PCR Clean-up kit (Macherey-Nagel, 740609) according to the manufacturer’s instructions, and quantified with NanoPhotometer® N60 (IMPLEN). Amplicons were submitted to cleavage assay with all ribonucleoproteins in a 5 μL reaction volume containing 0.5 μL of 15 μmol/L Cas9-RNP and 1 μL of 50 ng/μL PCR product in Optimized Minimal Essential Medium (Opti-MEM; Gibco, 31985070). Cleavage assay was performed at 37°C for 1 hour and the reaction was stopped at 85°C for 5 minutes. *In vitro* CRISPR/Cas9 assays were resolved in 3% agarose gels.

**4. Intrauterine electroporation of Cas9-RNP complexes**

*In vivo* intrauterine electroporation was performed as previously described [1] with some modifications. *In vivo* experiments were conducted using mTmG mice, aged between 3 and 6 months. Following the assembly of sgRNA-Cas9 complexes for electroporation, RNPs were pooled at equimolar concentration, and surgical procedures were performed as described below. Control experiments were performed in parallel using Cas9-only or non-targeting RNPs, prepared and electroporated under identical conditions, to evaluate nonspecific or Cas9-related effects. Female mice were anesthetized with 2% isoflurane via inhalation and received intraperitoneal analgesia (Buprex) at a dose of 8 mL/kg. The animals were placed in a ventral position, and a midline incision was made through the skin and the abdominal wall. Uterine horns were exposed, and 3 μL of RNP pool, mixed with Fast Green FCF (Sigma-Aldrich, 68724) for visualization, were injected into one uterine horn using a 700 Series Microliter Syringe, 30 G (Hamilton, 80408). After injection, using a BTX830 square electroporator (BTX), 4 pulses of 50 V for 50 msec spaced by 950 msec were applied to the injected uterine horn. This protocol was repeated, opposing the orientation of the tweezers, and performed along the entire length of the uterine horn. The tweezers used were Platinum Tweezertrode, 5 mm diameter (BTX). The contralateral uterine horn was left untreated to serve as an internal control. Once electroporated, the uterine horns were returned to the abdominal cavity, and the incision was closed using a wound stapler, AutoClip System (Fine Scientific Tools, FST, 12020-09). As previously published [1], we performed transient and DNA-free *in vivo* CRISPR/Cas9 editing via intrauterine electroporation of RNPs, achieving efficient gene editing in the endometrial epithelium without observable stromal or myometrial damage, and no impact on fertility or off-target systemic toxicity [1]. Mice were sacrificed by cervical dislocation at different time points post-electroporation, depending on the experiment. Uteri were dissected and processed as required for each analytical procedure.

**5. Isolation and culture of epithelial endometrial cells**

Isolation of epithelial endometrial cells was performed as previously described [2]. In brief, mice were euthanized by cervical dislocation, and the uteri were dissected. Uterine horns were cut into 3 mm pieces and washed in Hank's Balanced Salt Solution (HBSS; Gibco, 14175-046). Uterine fragments were digested with 1 % trypsin (Gibco, 15090-046) in HBSS for 1 hour at 4°C and for 45 minutes at room temperature. Dulbecco’s modified Eagle’s medium (DMEM; Gibco, 41965-018) with 10 % of inactivated Fetal Bovine Serum (FBSi; Gibco, A52567-01) was added to stop trypsin reaction. Then, with the edge of a razor blade, epithelial sheets were squeezed out of the uterine fragments. Epithelial sheets were washed twice with Phosphate Buffered Saline (PBS) and resuspended in 1 mL of basal medium: (DMEM-F/12; Gibco, 11039-021) with 1 mmol/L sodium pyruvate (Gibco, 11360-070), 1% penicillin/streptomycin (Gibco, 15140-122), and 0.1% amphotericin B (Gibco, I15290-018). Epithelial sheets were mechanically disrupted in clusters of cells by pipetting 50 times with a 1 mL tip. Clusters were diluted in basal medium supplemented with 2% dextran-coated charcoal-stripped serum (DCC; Gibco, A33821-01) and plated into 96-well plates, black with micro-clear bottom (Greiner Bio One, 655077). When required, phase-contrast or fluorescence images were taken with an Eclipse Ts2R microscope (Nikon), and quantification of GFP-positive cells was performed using Fiji software (version 2.16).

**6. NGS-amplicon sequencing analysis**

Genomic DNA (gDNA) for NGS-amplicon sequencing analysis was obtained from fresh electroporated uteri, from frozen tumors, and from paraffin-embedded tissues. In all cases, gDNA extraction was carried out using the NucleoSpin Tissue (Macherey-Nagel, 740952). Sample preparation was performed according to the type of sample: for fresh electroporated tissue, uteri were dissected and opened longitudinally to expose epithelial cells; frozen tissues were cut into 5 mm pieces; and 4 slices of 3 μm from paraffin-embedded tissues were deparaffinized in xylol. Then, samples were digested with proteinase K for 3 hours at 37°C and the protocol for gDNA extraction was carried out following the manufacturer’s instructions. Genomic DNA was amplified by PCRs flanking the RNP-targeted region. Primer sequences and PCR conditions are specified in **Supplementary Table S6** and **Supplementary Table S7**. PCR amplicons were resolved in agarose gels, purified using the NucleoSpin Gel and PCR Clean-up kit, following the manufacturer’s instructions, and quantified with NanoPhotometer® N60. Next Generation Sequencing (NGS)-Amplicon sequencing was performed by Genewiz (Azenta Life Sciences). Raw Fastq sequences were submitted to bioinformatic analysis to determine the presence of indels using Crispresso2 [3,4] and Cas-Analyzer [5] online tools. Editing percentages for targeted genes across different samples were analyzed and normalized using GraphPad Prism (version 8.0.1). For each sample, the gene with the highest editing percentage was set to 100% and the gene with the lowest to 0%, with all other gene values linearly scaled between these two extremes. This normalization allowed for direct comparison of editing profiles between samples, independently of absolute editing frequency.

**7. *In situ* RNA hybridization and rolling circle amplification for detection of CRISPR/Cas9-induced mutations**

For the padlock probe design, a detailed graphic of the design is provided in **Supplementary Figure S2A**. In brief, each crRNA target was used for determining the mRNA-specific region; each sequence was split into 2 and integrated into the padlock backbone. All padlock probe sequences are detailed in **Supplementary Table S8** and were obtained from IDT as 5’-phosphorylated DNA oligos, a requirement for ligation. For sample preparation, multi-well plates containing fibroblasts or epithelial cells were fixed for 10 minutes with 4% paraformaldehyde (PFA) and, after three PBS washes, incubated in methanol at -20°C for at least 4 hours. Then, cells were post-fixed with 4% PFA and preconditioned with hybridization buffer, containing 6× Saline Sodium Citrate (SSC) and 10% formamide (Thermo Scientific, 205820010). Hybridization of the padlock probes was done at a final concentration of 200 nmol/L for each probe (if more than one), in hybridization buffer, for 10 minutes at 55°C and overnight at 45°C. Then, rolling circle amplification (RCA) primers (**Supplementary Table S9**) were hybridized to padlock probes at a concentration of 200 nmol/L for 1 hour at 45°C. Samples were washed three times with 20% formamide in 2× SSC and twice with PBS and 0.1% Tween20 (PBS-T). For padlock probes ligation, samples were incubated for 2 hours at 37°C with ligation mix, containing 50% glycerol, 1× T4 RNA ligase Buffer (New England Biolabs, NEB, B0216L), 10 μmol/L ATP (Thermo Fisher Scientific, R0441), 0.2 μg/μL Recombinant Albumin (NEB, B9200), 0.1 U/μL Ribolock RNase inhibitor (Thermo Fisher Scientific, EO0382) and 0.5 U/μL SplintR ligase (NEB, M0375L). To wash the samples, they were rinsed twice with PBS-T. RCA was performed for 2 hours at 42°C using 0.5 U/μL EquiPhi29 DNA polymerase (Thermo Fisher Scientific, A39391) in a reaction containing 1× EquiPhi29 buffer, 1 mmol/L dithiothreitol (DTT) and 1 mmol/L dNTPs. Again, samples were washed three times with 20 % formamide in 2X SSC and twice with PBS-T. The visualization of the RCA products was performed through hybridization of detection probes and/or fluorescent-labeled probes (**Supplementary Table S10**). Specific detection probes and fluorescent-labelled probes were prehybridized at 95°C for 2 minutes and cooled to room temperature at a final concentration of 200 nmol/L each. Then, samples were incubated with the mixture of hybridized probes and Hoechst, in hybridization buffer, for 2 hours at 45°C. Finally, non-hybridized probes were removed with three wash steps of 20% formamide in 2X SSC and two of PBS-T. Samples were mounted in aqueous mounting medium Fluoromount-G (Thermo Fisher Scientific, 00-4958-02). Visualization and image acquisition were performed in EVOS FL inverted fluorescence microscope (Invitrogen), for generic probes detection, or with AX NSPARK confocal microscope (Nikon), for fluorochrome code detection.

**8. Image processing and RNA spots quantification**

Multichannel *.nd2 files, obtained from AX NSPARK confocal microscope, were converted to *.tiff files, without compression or scaling. Cell segmentation and spot segmentation for each channel (Atto488, Cy3, Atto647 or AlexaFluor750) were performed in CellProfiler (version 4.2.6) [6], using standard pipelines to identify nuclei, cell outlines and RNA spots. The output was exported as *.csv files, with individual spot positions and associated parent cells. Downstream analysis was performed in R (version 4.5.1) [7] using the tidyverse package [8]. Raw *.csv files containing spot data for each channel were imported, and cell identifiers were mapped. A threshold of 8 pixels was used as the maximum distance for defining spot colocalization between channels within the same cell. For each channel pair, the script calculates the number of colocalized spots per cell, using Euclidean distances between spot coordinates. Counting was performed for individual (non-colocalized) spots per channel, as well as for colocalized spots for each channel pair. Genes were assigned to single channels or colocalizations according to probe design. The final quantification table per cell includes individual and colocalization counts for all gene targets. For visualization of gene expression, spot counts were binarized (detection = 1 if at least one spot was detected, otherwise 0) and a binary heatmap was generated with the pheatmap package [9]. The complete R script, including data preprocessing, colocalization, and heatmap generation, is available on request.

**9. Tissue processing and immunohistochemistry analysis on paraffin sections**

For histological analysis, mice were euthanized, and uteri were dissected, formalin-fixed overnight at 4°C and paraffin-embedded. Paraffin sections of 3 μm were dried for 1 hour at 80°C, dewaxed in xylene, gradually rehydrated in ethanol and washed in PBS. Antigen retrieval was performed in EnVision FLEX high pH Solution (DAKO, K8004) for 20 minutes at 95°C. Then, samples were incubated with 3% H_2_O_2_ for endogenous peroxidase blocking, and washed three times with PBS. Primary antibodies were incubated for 30 minutes at room temperature, washed with PBS, and incubated with Horseradish Peroxidase (HRP)-conjugated or biotin-conjugated secondary antibodies and streptavidin-HRP, depending on the primary antibody. Finally, staining was visualized through reaction with EnVision Detection kit (DAKO, K4065) using diaminobenzidine (DAB) substrate. Slides were counterstained with Harris hematoxylin. Primary and secondary antibodies used for immunohistochemistry, and their dilutions, are detailed in **Supplementary Table S11**. When required, quantification of positive cells or positive nuclei in immunohistochemically stained sections was performed using QuPath (version 0.5.1) [10]. For nuclear markers (p53), automated cell detection algorithms were employed to identify and classify nuclei based on stain intensity. Intensity thresholds were established to segment nuclei into categories (negative, weak, moderate and strong) according to DAB chromogen signal, Histoscore (H-Score) quantification was subsequently calculated by combining the percentage of nuclei in each intensity category using the formula: H-score = (1 × % weakly positive) + (2 × % moderately positive) + (3 × % strongly positive), resulting in a value ranging from 0 to 300. For cellular markers (GFP or p16), positive cell detection was optimized for nuclear or cellular localization and exported as the percentage of positive cells. For both p53 and p16, a normal endometrial tissue sample was analyzed as a reference. All analyses were visually validated, and thresholding parameters were kept consistent across samples to allow quantitative comparison.

**10. Shallow WGS**

Genomic DNA (gDNA) from frozen tissues was extracted as previously described (see ***NGS-Amplicon Sequencing Analysis***). Shallow Whole Genome Sequencing (sWGS) was performed by Genewiz (Azenta Life Sciences). Raw FASTQ files were quality-checked using the fastqcr package in R [7, 11]. Reads were aligned to GRCm38/mm10 using BWA-MEM (v0.7.17) [12]. PCR duplicates were marked with the MarkDuplicates tool from GATK (v4.3.0.0) [13]. Genome-wide coverage profiles were generated with readCounter from the HMMcopy suite (v0.99.0) using a fixed bin size of 1 Mb [14]. Copy number alterations (CNAs) were estimated from sWGS using the ichorCNA algorithm [15]. CNA data generated by ichorCNA were imported into R and represented as GRanges and RaggedExperiment objects. Protein-coding gene annotations for Mus musculus were retrieved from Ensembl (release 100) using the AnnotationHub package [16].

**11. Statistical analysis**

Statistical analyses were performed using appropriate tests according to the type and design of each experiment, as detailed in each Method’s section. Specific statistical tests, sample sizes, and the number of independent experiments are described in the corresponding figure legends. Graphs and most statistical tests were generated using R (version 4.5.1) [7] and GraphPad Prism (version 8.0.1).

**Supplementary Tables**

**Supplementary Table S1.** Transfection methods for *in vivo* CRISPR/Cas9 delivery [1,17].

| Delivery system | Type | Type of CRISPR/Cas9 cargo | Advantages | Limitations |
| --- | --- | --- | --- | --- |
| AAV | Viral vector | Cas9 DNA + sgRNA | - High efficiency - Stable expression | - Immunogenicity - Long term Cas9 expression |
| EP | Physical | RNP | - Immediate activity - Multiplexable - No DNA integration - Defined temporal control | - Medium efficiency |
| EV | Natural nanoparticle | Cas9 mRNA + sgRNA  RNP | - Biocompatible - Low immunogenicity - Non-viral | - Low cargo loading efficiency - Limited control of biodistribution |
| LNP | Synthetic vesicle | Cas9 mRNA + sgRNA  RNP | - Transient expression - Scalable | - Limited tissue targeting - Biased biodistribution - Immunogenicity |
| PNP | Synthetic polymer | Cas9 mRNA + sgRNA  RNP | - Chemically tunable - Cationic - Localized delivery | - Low efficiency - Cytotoxic - Limited data |

**Abbreviations:** AAV: adeno-associated virus; EP: electroporation; EV: extracellular vesicles; LNP: lipid nanoparticles; PNP: polymeric nanoparticles.

**Supplementary Table S2**. Genotyping primers and PCR conditions.

| **Allele** | **Primers (5’ to 3’)** | | **PCR conditions** | | | **PCR product** | |
| --- | --- | --- | --- | --- | --- | --- | --- |
|  |  |  | **Temp. (°C)** | **Time** | **Cycles** |  |  |
| **Cre:ER^(T)^** | Fwd: | ACGAACCTGGTCGAAATCGTGCG | 94 | 2 min | 1 | Cre:ER^T-/-^ | No band |
|  |  |  | 94 | 45 sec | 32 |  |  |
|  |  |  | 65 | 45 sec |  |  |  |
|  | Rev: | CGGTCGATGCAACGAGTGATGAG | 72 | 45 sec |  | Cre:ER^T+/-^ | 350 bp |
|  |  |  | 72 | 5 min | 1 |  |  |
|  |  |  | 4 | ∞ | 1 |  |  |
| ***Pten*^f/f^** | Fwd: | CAAGCACTCTGCGAACTGAG | 94 | 3 min | 1 | *Pten*^+/+^ | 156 bp |
|  |  |  | 94 | 30 sec | 35 |  |  |
|  |  |  | 60 | 1 min |  | *Pten*^f/+^ | 156 bp & 328 bp |
|  | Rev: | AAGTTTTTGAAGGCAAGATGC | 72 | 2 min |  |  |  |
|  |  |  | 72 | 2 min | 1 | *Pten*^f/f^ | 328 bp |
|  |  |  | 4 | ∞ | 1 |  |  |
| ***mTmG*** | Com: | CTCTGCTGCCTCCTGGCTTCT | 94 | 2 min | 1 | *mTmG*^+/+^ | 330 bp |
|  |  |  | 94 | 30 sec | 35 |  |  |
|  | Wt: | CGAGGCGGATCACAAGCAATA | 59 | 30 sec |  | *mTmG*^f/+^ | 250 bp & 330 bp |
|  |  |  | 72 | 2 min |  |  |  |
|  | Mut: | TCAATGGGCGGGGGTCGTT | 72 | 5 min | 1 | *mTmG*^f/f^ | 250 bp |
|  |  |  | 4 | ∞ | 1 |  |  |

**Abbreviations:** bp: base pairs; Com: common primer; Cre:ER^(T)^: tamoxifen-induclible Cre (fused to modified estrogen receptor); f/f: floxed/floxed; Fwd: forward primer; min: minutes; mTmG: membrane-targeted tandem dimer Tomato/membrane-targeted Green Fluorescent Protein; Mut: mutant reverse primer; Rev: reverse primer; sec: seconds; Temp.: temperature; Wt: wild-type reverse primer.

**Supplementary Table S3**. Sequence of crRNA targets used for CRISPR/Cas9 gene editing.

| Target gene | crRNA target^*^ (5’ to 3’) |
| --- | --- |
| mTmG *loxP* | GTATGCTATACGAAGTTATTAGG |
| *Fbxw7* (exon 5) | GTTGTTGGTGTTGCTGAACATGG |
| *Pten* (exon 5) | AATTCACTGTAAAGCTGGAAAGG |
| *Trp53* (exon 7) | GGAGTCTTCCAGTGTGATGATGG |
| *Ppp2r1a* (exon 5) | TGTCATCTGAGCACAGGTTCCGG |
| *Arhgap35* (exon 1) | AAGACTTCCCAATGCCGCACTGG |
| *Arid1a* (exon 1) | AAGAACTCGAACGGGAACGCGGG |
| *Pik3r1* (exon 1) | CTTACGTTGAATACATTGGAAGG |
| *Muc16* (exon 2) | ATAAAGAGGGCTTCTCGTCAGGG |
| *Kmt2d* (exon 4) | ACTGCCAACTGGCACGCTTGCGG |
| *Chd4* (exon 2) | TCGGACCCTCACCAACTACAAGG |

^*^Underlined nucleotides represent the PAM sequence. **Abbreviations:** *Arhgap35*: rho GTPase activating protein 35; *Arid1a*: AT-rich interaction domain 1A; *Chd4*: chromodomain-helicase-DNA-binding protein 4; *Fbxw7*: F-box and WD repeat domain containing 7; *Kmt2d*: lysine methyltransferase 2D; mTmG: membrane-targeted tandem dimer Tomato/membrane-targeted Green Fluorescent Protein*; Muc16*: mucin 16; PAM: protospacer adjacent motif; *Pik3r1*: phosphoinositide-3-kinase regulatory subunit; *Ppp2r1a*: protein phosphatase 2 scaffold subunit alpha; *Pten*: phosphatase and tensin homolog; *Trp53*: transformation related protein 53.

**Supplementary Table S4**. Sequence of DNA oligonucleotides used for CRISPR *in vitro* tandem assays. Amplification regions are marked in blue, and gene targets are separated with slashes.

| DNA sequence (5’ to 3’) | |
| --- | --- |
| CRISPR *tandem assay* 1 | Amp fwd**/***loxP***/***Fbxw7***/***Pten***/***Trp53***/***Ppp2r1a***/**Amp rev |
|  | GGAAGTGGCTCAGGTTCTGGA**/**GTATGCTATACGAAGTTATTAGG**/**AATTCACTGTAAAGCTGGAAAGG**/**GGAGTCTTCCAGTGTGATGATGG**/**TGTCATCTGAGCACAGGTTCCGG**/**CCATGTTCAGCAACACCAACAAC**/**CTCTGTATGCGATCGGCCAAG |
| CRISPR *tandem assay* 2 | Amp fwd**/***Arhgap35***/**Arid1a**/**Pik3r1**/**Kmt2d**/**Chd4**/**Amp rev |
|  | GGAAGTGGCTCAGGTTCTGGA**/**AAGACTTCCCAATGCCGCACTGG**/**AAGAACTCGAACGGGAACGCGGG**/**CTTACGTTGAATACATTGGAAGG**/**ATAAAGAGGGCTTCTCGTCAGGG**/**ACTGCCAACTGGCACGCTTGCGG**/**CCTTGTAGTTGGTGAGGGTCCGA**/**CTCTGTATGCGATCGGCCAAG |

**Abbreviations:** Amp fwd: forward amplifier; Amp rev: reverse amplifier.

**Supplementary Table S5**. Sequence of primers and PCR conditions for CRISPR tandem assay oligonucleotides amplification.

|  | **Primer sequence (5’ to 3’)** | | **PCR program** | | |
| --- | --- | --- | --- | --- | --- |
|  |  |  | **Temp. (°C)** | **Time** | **Cycles** |
| **CRISPR tandem assays 1, 2** | Fwd | AGAAGGAGATATAACTGGAAGTGGCTCAGGTTCTGGA | 96 | 1 min | 1 |
|  |  |  | 96 | 10 sec | 30 |
|  |  |  | 65 | 20 sec |  |
|  | Rev | GGAGATGGGAAGTCACTTGGCCGATCGCATACAGAG | 72 | 30 sec |  |
|  |  |  | 72 | 10 min | 1 |
|  |  |  | 4 | ∞ | 1 |

**Abbreviations:** Fwd: forward; min: minutes; Rev: reverse; sec: seconds; Temp: temperature.

**Supplementary Table S6**. Sequence of primers used for NGS-amplicon sequencing.

| Target gene (product size) | Forward primer (5’ to 3’) | Reverse primer (5’ to 3’) |
| --- | --- | --- |
| *Fbxw7* (218 bp) | ATACATCTGGGGCAAGCTTAAGGTCTTACGTATAAGCAGG | CTGAAACATTTTCAGCCACTCTTGAAGGCCTGTGGGTGGT |
| *Pten* (230 bp) | ATCCTTTTGAAGACCATAACCCACCACAGCTAGAACTTAT | CTTTTTGTCTCTGGTCCTTACTTCCCCATAAAAATCTAGG |
| *Trp53* (220 bp) | GCCGGCTCTGAGTATACCACCATCCACTACAAGTACATGT | TAGGAACCAAAGAGCGTTGGGCATGTGGTAGGGGG |
| *Ppp2r1a* (225 bp) | AGTGCTCTGTGCTGTGCCGGGACACTAGCAGTTAGGACTT | CTGCTCGTCAGAGGCCAGGTTAGAGAACATGGGAATGATC |
| *Arhgap35* (245 bp) | ATGATGATGGCAAGAAAGCAAGATGTCCGCATCCCCACCT | GAGCGGCTAACTTCTCCCCAGTACAGAAAGTGGTCATTAT |
| *Arid1a* (479 bp) | CTCGGAGCTGAAGAAAGCCGAGCAGCAGCAGCGGGAGGA | GGCCAGGGCTTTGTTGTCCGCCATGTTGTTGGTGGAAGA |
| *Pik3r1* (200 bp) | CCAGGAAGCCCGGCCTGAAGATATTGGCTGGTTAAATGGC | CTTGCTGCTCCGTGTCAGCTTCAGTTTTTGAAGAACCCGG |
| *Muc16* (240 bp) | GAATTTTCACAGACAAGCTCTGCTTCCTCTGTTAACTCAG | ATTAGCTGTTCCAAAGATGGAGGTTAGCCCTATGTGTTCT |
| *Kmt2d* (235 bp) | AGGAGGCTCGCTGTGCAGTGTGTGAGGGGCCAGGGCAGCT | CAGGACACATTGTGGGATCCTTCCTAGCCTCATTTTCTGTCCACAC |
| *Chd4* (256 bp) | TTAGAAGACTGGGGTATGGAAGACATCGACCATGTGTTCT | ATCTTGGAGACAGCAATCTTGGGGTTTTTAGCAGCAATCA |

**Abbreviations:** *Arhgap35*: rho GTPase activating protein 35; *Arid1a*: AT-rich interaction domain 1A; bp: base pair; *Chd4*: chromodomain-helicase-DNA-binding protein 4; *Fbxw7*: F-box and WD repeat domain containing 7; *Kmt2d*: lysine methyltransferase 2D*; Muc16*: mucin 16; *Pik3r1*: phosphoinositide-3-kinase regulatory subunit; *Ppp2r1a*: protein phosphatase 2 scaffold subunit alpha; *Pten*: phosphatase and tensin homolog; *Trp53*: transformation related protein 53.

**Supplementary Table S7**. PCR conditions used for NGS amplicon sequencing.

| **PCR program** | | |
| --- | --- | --- |
| **Temp. (°C)** | **Time** | **Cycles** |
| 95 | 5 min | 1 |
| 95 | 15 sec | 45 |
| 72 | 30 sec |  |
| 72 | 5 min | 1 |
| 4 | ∞ | 1 |

**Abbreviations:** *min: minutes; sec: seconds; Temp: temperature.*

**Supplementary Table S8.** Sequence of padlock probes used for RCA experiments.

| Target gene | Padlock probe sequence^*^ (5’ to 3’) |
| --- | --- |
| *Fbxw7* | /5Phos/ACATGGTACAAGGCCAGTGGTTGACGTATATCTTGCGAGTGAGCGACCTCAATGCTGCTGCTGTACTACTAAAGTTGTTGGTGTTGCTGA |
| *Pten* | /5Phos/CAGCTTTACAGTGAATTGCTGCTGACGTATATCTTGCGAGTGAGCGACCTCAATGCTGCTGCTGTACTACTACACCAGTCCGTCCCTTTC |
| *Trp53* | /5Phos/TGATGGTAAGGATAGGTCGGCCTGACGTATATCTTGCGAGTGAGCGACCTCAATGCTGCTGCTGTACTACTCTGGAGTCTTCCAGTGTGA |
| *Ppp2r1a* | /5Phos/TTCCGGAAGTACTGTCGAAGCTGACGTATATCTTGCGAGTGAGCGACCTCAATGCTGCTGCTGTACTACTGGGTGTCATCTGAGCACAGG |
| *Arhgap35* | /5Phos/ACTGGCCTTTCTCCTTCTCACTGACGTATATCTTGCGAGTGAGCGACCTCAATGCTGCTGCTGTACTACTACAAGACTTCCCAATGCCGC |
| *Arid1a* | /5Phos/TTCCCGTTCGAGTTCTTCAGCTGACGTATATCTTGCGAGTGAGCGACCTCAATGCTGCTGCTGTACTACTGGGCGGGCCTAGGGCCCGCG |
| *Pik3r1* | /5Phos/AATGTATTCAACGTAAGTTCCTGACGTATATCTTGCGAGTGAGCGACCTCAATGCTGCTGCTGTACTACTGGTGAAATTCTTTTCCTTCC |
| *Muc16* | /5Phos/TCAGGGTTCTGCCAGCTCTACTGACGTATATCTTGCGAGTGAGCGACCTCAATGCTGCTGCTGTACTACTGGAATAAAGAGGGCTTCTCG |
| *Kmt2d* | /5Phos/TTGCGGGCAGTCAGAGCAGTCTGACGTATATCTTGCGAGTGAGCGACCTCAATGCTGCTGCTGTACTACTGGCACTGCCAACTGGCACGC |
| *Chd4* | /5Phos/AGTTGGTGAGGGTCCGATAACTGACGTATATCTTGCGAGTGAGCGACCTCAATGCTGCTGCTGTACTACTAAATTGGCTGAAGGCCTTGT |
| *Actb* | /5Phos/AGCACTTGCGGTGCACGATGCTGACGTATATCTTGCGAGTGAGCGACCTCAATGCTGCTGCTGTACTACTTCAGTAACAGTCCGCCTAGA |

^*^"/5Phos/" indicates phosphorylation at the 5’ end. **Abbreviations:** *Actb*: actin b*; Arhgap35*: rho GTPase activating protein 35; *Arid1a*: AT-rich interaction domain 1A; *Chd4*: chromodomain-helicase-DNA-binding protein 4; *Fbxw7*: F-box and WD repeat domain containing 7; *Kmt2d*: lysine methyltransferase 2D*; Muc16*: mucin 16; *Pik3r1*: phosphoinositide-3-kinase regulatory subunit; *Ppp2r1a*: protein phosphatase 2 scaffold subunit alpha; *Pten*: phosphatase and tensin homolog; *Trp53*: transformation related protein 53.

**Supplementary Table S9.** Sequence of RCA primers used in RCA experiments.

| Primer | Primer sequence |
| --- | --- |
| RCA 1 | ACAGCAGCAGCATTGAGG*T*C |
| RCA 2 | CGCAAGATATACG*T*C |

Asterisks indicate phosphorothioate bonds. **Abbreviations:** RCA 1: rolling circle amplification primer 1; RCA 2: rolling circle amplification primer 2.

**Supplementary Table S10.** Sequence of detection probes used for visualization of RCA experiments.

| Target site (probe) | Probe sequence (5’ to 3’)^*^ |
| --- | --- |
| RCA 1 | /FAM/CCTCAATGCTGCTGCTGTACTAC |
| RCA 2 | /FAM/CTGACGTATATCTTGCGAGTGAG/FAM/ |
| Cy3*-T3*-Cy3 | /Cy3/GCGCGAAATTAACCCTCACTAAAGG/Cy3/ |
| Atto488 | /ATTO488/TCCGAGTACGCT |
| Cy3 | /Cy3/AGTCCGTAGCGA |
| Atto647 | /ATTO647/CGTACGCTAGTC |
| AlexaFluor750 | /AF750/TGCAGTCCGATC |
| *Actb-T3* | GTAACAGTCCGCCTAGAAGCACTTGCGGTGCACGACCTTTAGTGAGGGTTAATTTCGCGC |
| *Actb-*4 color | GATCGGACTGCATCGCTACGGACTGTAACAGTCCGCCTAGAAGCACTTGCGGTGGACTAGCGTACGAGCGTACTCGGA |
| *Fbxw7*-Atto488 | TGTTGGTGTTGCTGAACATGGTACAAGGCCAGCGTACTCGGA |
| *Pten*-Atto647 | AGTCCGTCCCTTTCCAGCTTTACAGTGAATGACTAGCGTACG |
| *Trp53*-Cy3 | AGTCTTCCAGTGTGATGATGGTAAGGATAGTCGCTACGGACT |
| *Ppp2r1a*-AF750 | TCATCTGAGCACAGGTTCCGGAAGTACTGTGATCGGACTGCA |
| *Arhgap35*-Cy3/Atto647 | GACTAGCGTACGACTTCCCAATGCCGCACTGGCCTTTCTCCTTCGCTACGGACT |
| *Arid1a*-Atto488/Cy3 | AGCGTACTCGGAGGCCTAGGGCCCGCGTTCCCGTTCGAGTTCTCGCTACGGACT |
| *Pik3r1*-Atto488/AF750 | AGCGTACTCGGAAATTCTTTTCCTTCCAATGTATTCAACGTAGATCGGACTGCA |
| *Muc16*-Cy3/AF750 | TCGCTACGGACTAAAGAGGGCTTCTCGTCAGGGTTCTGCCAGGATCGGACTGCA |
| *Kmt2d*-Atto488/Atto647 | GACTAGCGTACGTGCCAACTGGCACGCTTGCGGGCAGTCAGAAGCGTACTCGGA |
| *Chd4*-Atto647/AF750 | GACTAGCGTACGGGCTGAAGGCCTTGTAGTTGGTGAGGGTCCGATCGGACTGCA |

^*^Codes between slashes represent FAM, Cy3, ATTO488, ATTO647 or AlexaFluor (AF)750 labelling. respectively. **Abbreviations:** *Actb*: actin b*; Arhgap35*: rho GTPase activating protein 35; *Arid1a*: AT-rich interaction domain 1A; *Chd4*: chromodomain-helicase-DNA-binding protein 4; *Fbxw7*: F-box and WD repeat domain containing 7; *Kmt2d*: lysine methyltransferase 2D*; Muc16*: mucin 16; *Pik3r1*: phosphoinositide-3-kinase regulatory subunit; *Ppp2r1a*: protein phosphatase 2 scaffold subunit alpha; *Pten*: phosphatase and tensin homolog; RCA 1: rolling circle amplification detection primer 1; RCA 2: rolling circle amplification detection primer 2; *Trp53*: transformation related protein 53; T3: T3 promoter sequence; T7: T7 promoter sequence.

**Supplementary Table S11.** Antibodies and reagents used in immunohistochemistry analysis.

| Product name | Dilution | Catalogue number, company | Antigen retrieval | Secondary antibody |
| --- | --- | --- | --- | --- |
| Anti-β-catenin | RTU | IR702, Dako | pH 9 | EV Flex Kit |
| Anti- Cytokeratin 8 | RTU | AB531826, DSHB | pH 9 | Rabbit-biotin |
| Anti-GFP | 1:100 | 600-101-215, Rockland | pH 9 | Goat-biotin |
| Anti-p16 | RTU | CNIO Antibodies Core Unit | Unknown | Unknown |
| Anti-p53 | RTU | CNIO Antibodies Core Unit | Unknown | Unknown |
| Anti-Vimentin | 1:500 | ab92547, Abcam | pH 9 | Rabbit-biotin |
| Anti-Goat IgG-biotin | 1:200 | SC-2489, Santacruz | NA | NA |
| Anti-Rabbit IgG-biotin | 1:200 | 111-065-144, Jackson | NA | NA |
| Streptavidin-HRP | 1:400 | P0397, Dako | NA | NA |
| EnVision Flex Detection Kit | RTU | K8002, Dako | NA | NA |

**Abbreviations:** HRP: horseradish peroxidase; NA: not applicable; RTU: ready to use.

**Supplementary Figures**

**
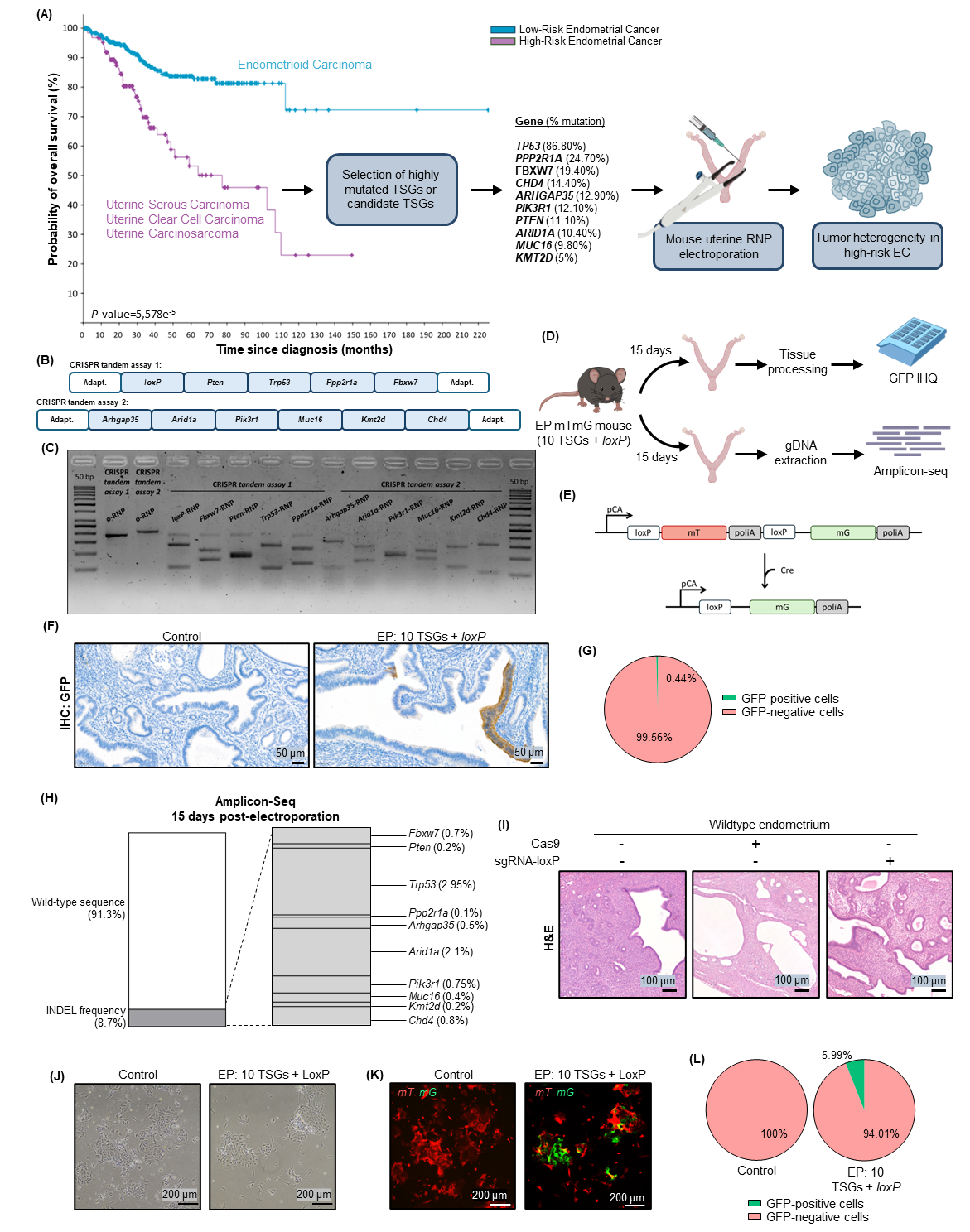
**

**Supplementary Figure S1. CRISPR/Cas9 editing of frequently mutated tumor suppressor genes in high-risk endometrial cancer. (A)** Illustration of the experimental design: Kaplan-Meier curve, comparing overall survival from low-risk endometrial cancer subtypes (endometrioid carcinoma) and high-risk endometrial cancer subtypes (Uterine Serous Carcinoma, Uterine Clear Cell Carcinoma, Uterine Carcinosarcoma), obtained from cBioPortal [18–20] with a *P* value = 5.578e^-5^. The most frequently mutated TSGs, or candidate TSGs (genes that have not yet been validated as TSGs), in high-risk endometrial cancer were identified using cBioPortal and validated through OncoKB. RNP complexes targeting the selected TSGs were electroporated into the uterine cavity to induce tumor heterogeneity. **(B)** Representative diagram of DNA templates used for *in vitro* CRISPR/Cas9 tandem assay. **(C)** Representative image of an agarose-resolved *in vitro* CRISPR/Cas9 digestion of all RNP target sequences. **(D)** Diagram of the experimental design for validation of *in vivo* CRISPR/Cas9-mediated gene editing. **(E)** Structure of the pCA-mTmG cassette, where pCA is Cytomegalovirus enhancer and chicken β-Actin promoter; polyA represent polyadenylation signals; mT encodes for the fluorescent protein tdTomato and mG the fluorescent protein GFP. **(F)** Representative image of GFP IHC of non-electroporated (control) and electroporated with a pool of RNPs (EP: 10 TSGs + *loxP*). **(G)** Quantification of GFP-positive cells in samples shown in *(C)*. **(H)** Percentages of edited sequences of CRISPR/Cas9-targeted tumor suppressor genes obtained in Amplicon-Seq analysis. **(I)** H&E staining of uterine sections 16 weeks after electroporation of Cas9 alone or sgRNA alone. **(J)** Representative phase-contrast images of non-electroporated (control) and electroporated with a pool of RNPs (EP: 10 TSGs + *loxP*) epithelial endometrial cells 48h after being cultured. **(K)** Representative fluorescence images of non-electroporated (control) and electroporated with a pool of RNPs (EP: 10 TSGs + *loxP*) epithelial endometrial cells 48h after being cultured. **(L)** Quantification of GFP-positive cells in samples shown in *(J)*. **Abbreviations**:  *Arhgap35*: rho GTPase activating protein 35; *Arid1a*: AT-rich interaction domain 1A; *Chd4*: chromodomain-helicase-DNA-binding protein 4; EP: electroporated; *Fbxw7*: F-box and WD repeat domain containing 7; GFP: green fluorescent protein; H&E: hematoxylin and eosin; IHC: immunohistochemistry; *Kmt2d*: lysine methyltransferase 2D; *Muc16*: mucin 16; *Pik3r1*: phosphoinositide-3-kinase regulatory subunit; *Ppp2r1a*: protein phosphatase 2 scaffold subunit alpha; *Pten*: phosphatase and tensin homolog; RNP: ribonucleoprotein; *Trp53*: transformation related protein 53; TSG: tumor suppressor gene.

**
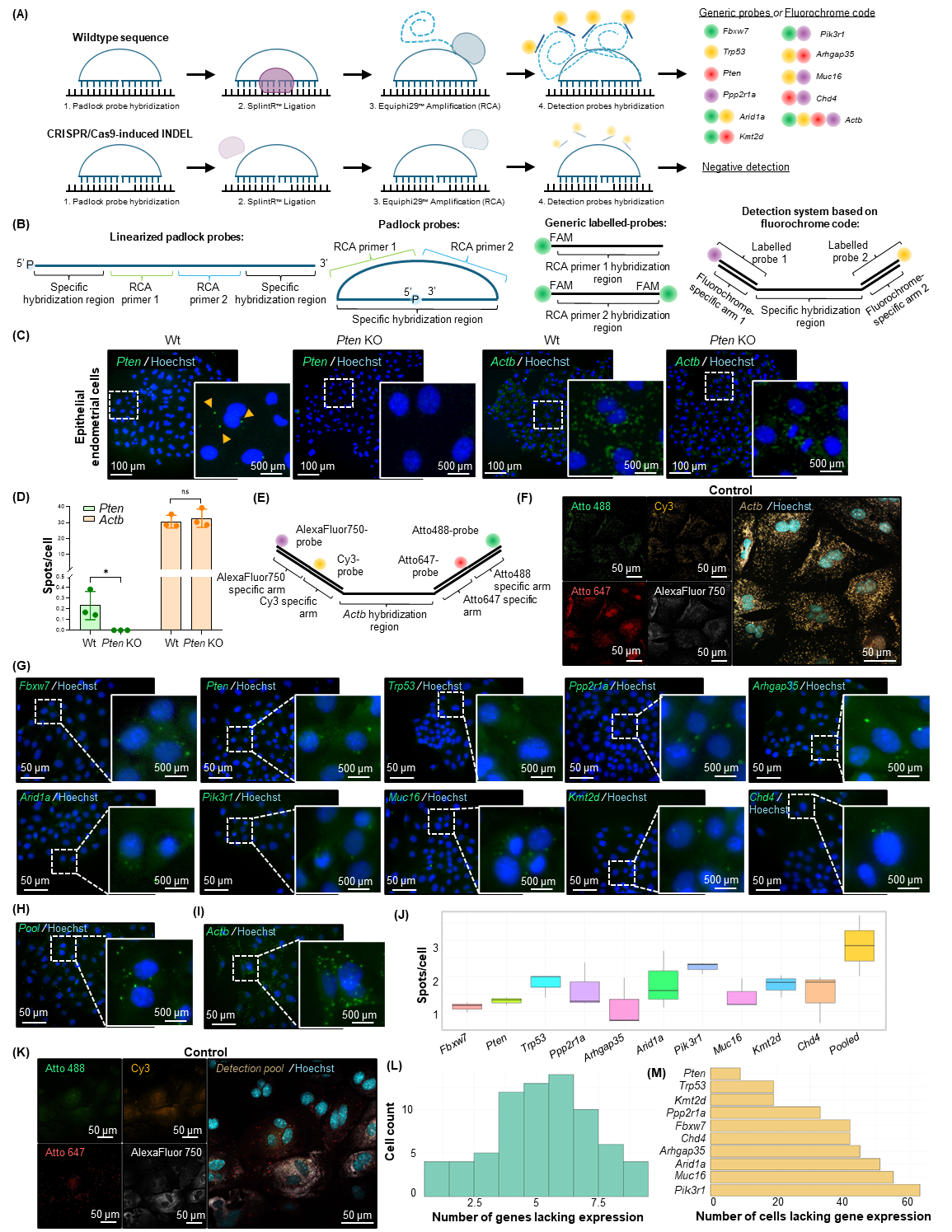
**

**Supplementary Figure S2. Intrauterine delivery of the CRISPR/Cas9 system against the most frequently mutated tumor suppressor genes in EC leads to heterogeneous populations of epithelial endometrial cells.** **(A)** Graphic representation of the spatial mRNA-based method used for validation of tumor heterogeneity generated with CRISPR/Cas9. Briefly, a padlock probe is hybridized in the mRNA of the specific gene, designed at the Cas9-cutting site. Then, SplintR ligase is used to ligate and circularize the padlock probe, but if there is not 100% homology between the padlock probe and the mRNA (edited sequence), ligation does not occur. The next step is RCA, where EquiPhi29 polymerase amplifies the padlock sequence. Finally, specific regions of different padlock probes are detected with fluorochrome-labelled probes. If the detection is individual for a single gene, generic probes are used. But if the detection is pooled, a fluorochrome code is used. **(B)** Illustration of the design of probes used for *in situ* RNA detection. Padlock probes, phosphorylated (P) at the 5’ end, contain complementary regions of RCA primers 1 and 2 flanked by the specific hybridization region, complementary to the sgRNA target site. Generic detection probes are complementary to the RCA product at the padlock backbone and are labelled at 5’ or at both 5’ and 3’ ends with FAM fluorochromes. Probes for the detection with fluorochrome code have a specific hybridization region to each gene and 1 or 2 arms complementary to the labelled probes. Labelled probes are marked with a molecule of Atto488, Cy3, Atto647 or AlexaFLuor750 at their 5’ end. **(C)** Representative images of *Pten* (green, left) or *Actb* detection (green, right) in epithelial endometrial cells from Cre:ER^T-/-^ *Pten*^F/F^ (Wt) and Cre:ER^T+/-^ *Pten*^F/F^ (*Pten* KO) mice. Orange arrows show *Pten* mRNA spots. Hoechst (blue) is used for nuclear staining. **(D)** Spot quantification of samples is shown in the left panel. Data is represented as the mean of spots/cell ± s.e.m. **(E)** Illustration of the design of *Actb* detection system with 4 color-labelled probes. **(F)** *Actb* mRNA detection with the 4-color probe, containing arms for all labelled probes, as represented in the lower panel. Images show detection with Atto488 (green), Cy3 (orange), Atto647 (red), and/or AlexaFluor750 (grey). Hoechst (blue) is used for nuclear staining. **(G)** Individual detection of selected TSGs (*Fbxw7, Pten, Trp53, Ppp2r1a, Arhgap35, Arid1a, Pik3r1, Muc16, Kmt2d,* and *Chd4*) in wild-type epithelial endometrial cells detected with generic probes (green). Hoechst (blue) is used for nuclear staining. **(H)** Pooled detection of mRNA from selected TSGs in wild-type epithelial endometrial cells detected with generic probes (green). Hoechst (blue) is used for nuclear staining. **(I)** Individual detection of *Actb* mRNA with generic probes. **(J)** Distribution of spots per cell across different gene perturbations. Box-and-whisker plots show the number of spots per cell for each targeted gene (*Fbxw7, Pten, Trp53, Ppp2r1a, Amigo3, Arid1a, Pik3r1, Muc16, Kmt2d,* and *Chd4*), as well as for the pooled condition. Boxes represent the interquartile range, the horizontal line indicates the median, and whiskers denote the minimum and maximum values. The pooled condition displays the highest variability and overall number of spots per cell compared with individual gene perturbations. **(K)** Representative image of pooled detection of selected TSG in non-electroporated (control) epithelial endometrial cells, detected with fluorochrome code. Images show detection with Atto488 (green), Cy3 (orange), Atto647 (red) and/or AlexaFluor750 (grey). Hoechst (blue) is used for nuclear staining. **(L)** Histogram showing the distribution of the total number of genes lacking expression in EP:10 TSG across all analyzed cells. Each bar represents the count of cells exhibiting a given number of lost genes. **(M)** Bar plot displaying, for each analyzed gene, the number of cells showing loss of expression. **Abbreviations**: *Actb*: actin b; *Arhgap35*: rho GTPase activating protein 35; *Arid1a*: AT-rich interaction domain 1A; *Chd4*: chromodomain-helicase-DNA-binding protein 4; EP: electroporated; *Fbxw7*: F-box and WD repeat domain containing 7; *Kmt2d*: lysine methyltransferase 2D; *Muc16*: mucin 16; *Pik3r1*: phosphoinositide-3-kinase regulatory subunit; *Ppp2r1a*: protein phosphatase 2 scaffold subunit alpha; *Pten*: phosphatase and tensin homolog; RCA: rolling circle amplification; s.e.m.: standard error of the mean; *Trp53*: transformation related protein 53; TSG: tumor suppressor gene.

**
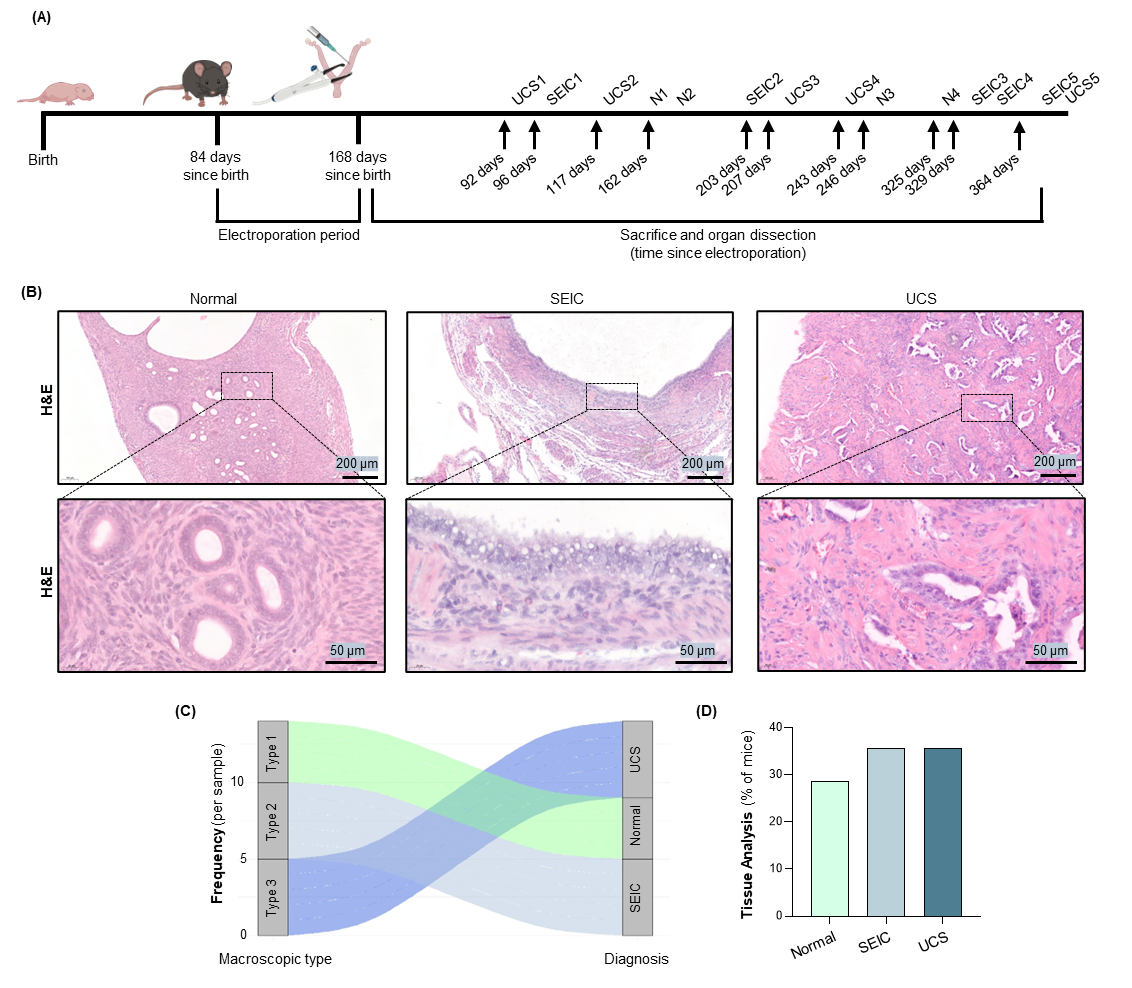
**

**Supplementary Figure S3. Intrauterine deletion of tumor suppressor genes leads to the formation of high-risk endometrial cancer subtypes. (A)** Timeline for the *in vivo* analysis of endometrial lesions in electroporated mice (*n* = 14). **(B)** Representative H&E staining of lesion groups: normal, SEIC, and UCS from endometrial sections of EP mice. **(C)** Histopathological analysis of endometrial sections shown in *(B)*. Type 1 lesions correspond to normal (N) histology, while types 2 and 3 correspond to SEIC and UCS, respectively. **(D)** Percentages of each histological subtype. **Abbreviations**: EP: electroporated; H&E: hematoxylin and eosin; N: normal; SEIC: serous endometrial intraepithelial carcinoma; UCS: uterine carcinosarcoma.


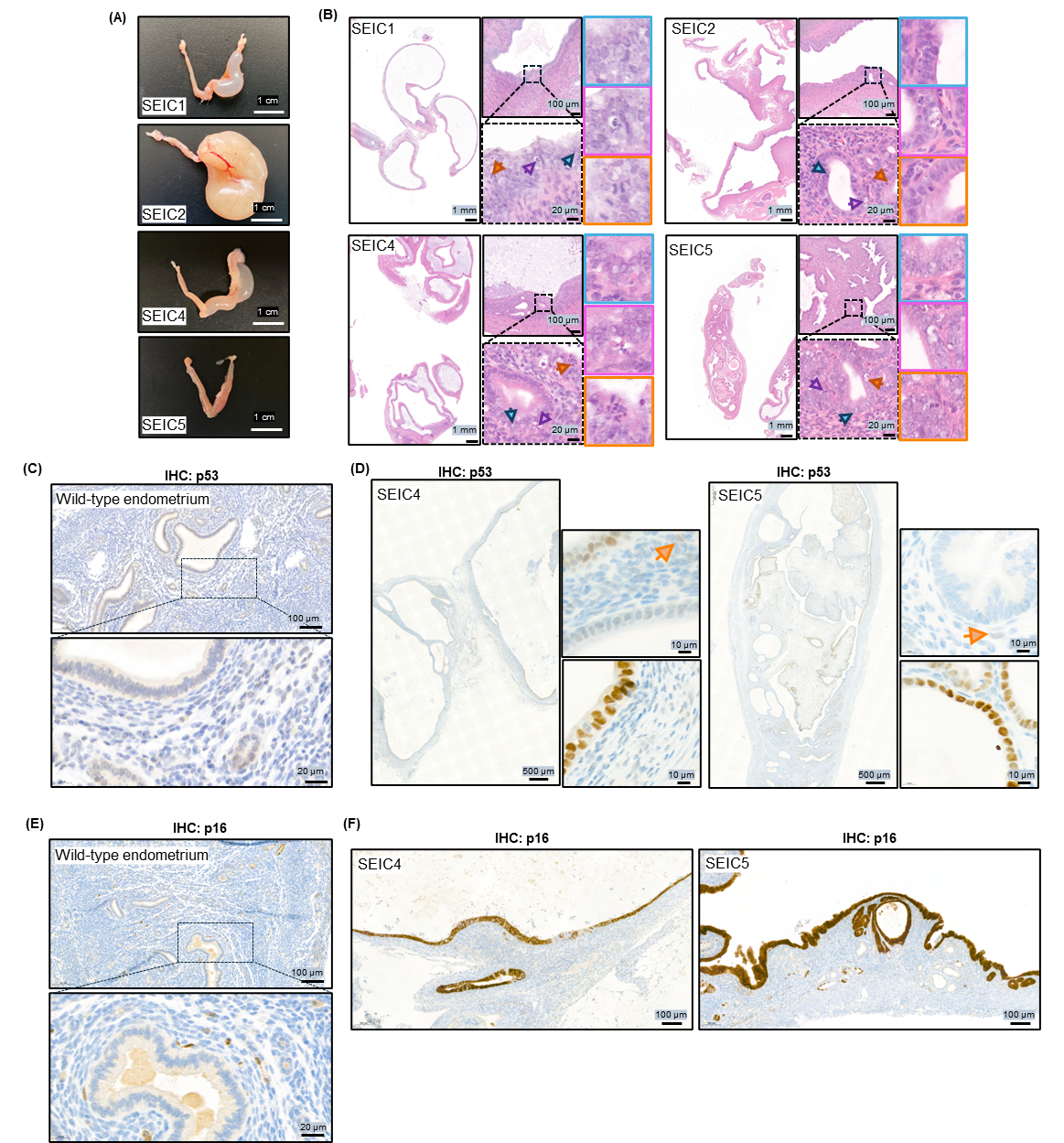


**Supplementary Figure S4. SEICs display molecular features of human ECs. (A)** Macroscopic images of uteri displaying SEIC (cases SEIC1, SEIC2, SEIC4, and SEIC5). **(B)** H&E staining of cases SEIC1, SEIC2, SEIC4 and SEIC5. Blue arrows and squares show pleomorphic nuclei; purple arrows and squares show apoptotic cells, and orange arrows and squares show mitotic cells. **(C)** Representative image of p53 IHC of wild-type endometrium used as control for quantification of nuclear p53 and H-score in *Figure 5G*. **(D)** Representative image of p53 IHC of samples SEIC4 and SEIC5. **(E)** Representative image of p16 IHC of wild-type endometrium, used as control for quantification of cytoplasmic p16 in *Figure 5I*. **(F)** Representative images of p16 IHC of samples SEIC4 and SEIC5. **Abbreviations:** H&E: hematoxylin and eosin; IHC: immunohistochemistry, SEIC: serous endometrial intraepithelial carcinoma.


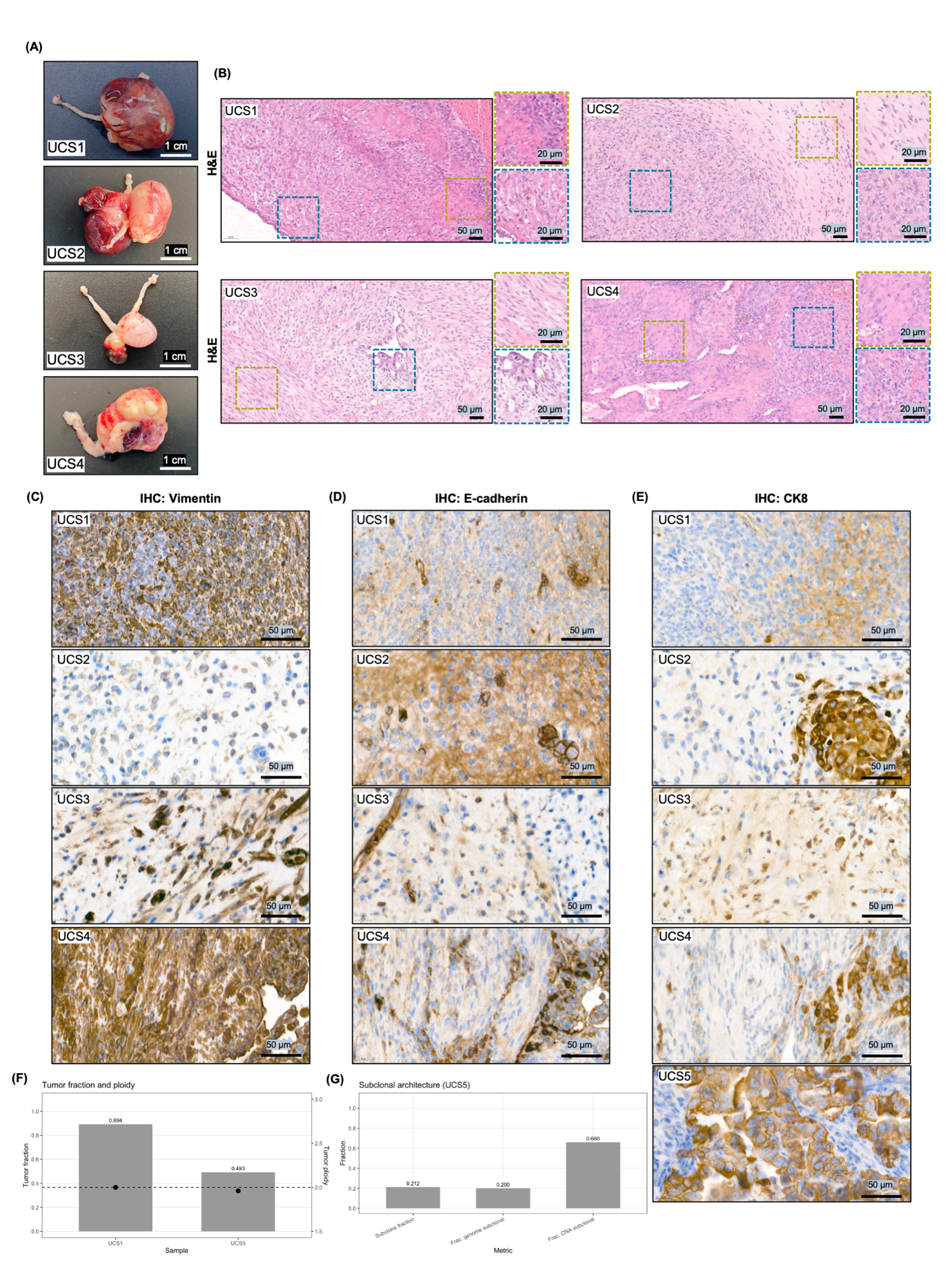


**Supplementary Figure S5. UCSs display molecular features of human EC. (A)** Macroscopic images of samples UCS1, UCS2, UCS3 and UCS4. **(B)** H&E representative images of cases UCS1, UCS2, UCS3 and UCS4. Green squares highlight the sarcomatous compartment, and blue squares denote the carcinomatous compartment. (**C-E**) Representative Vimentin (C), E-cadherin (D) and CK8 (E) IHCs of UCS1, UCS2, UCS3 and UCS4. **(F)** Tumor fraction and tumor ploidy estimated by ichorCNA for UCS1 and UCS5. Bars show the tumor fraction foe each sample (UCS1 = 0.8936; UCS5 = 0.4928), and overlaid points indicate the corresponding tumor ploidy (UCS1 = 2.0; UCS5 = 1.96), with the dashed horizontal line marking diploid ploidy (2.0). **(G)** Subclonal architecture metrics for UCS5. Bars display the subclone fraction (0.212; fraction of tumor DNA assigned to subclonal copy number states), the fraction of the genome affected by subclonal CNAs (Frac. Genome subclonal = 0.20), and the fraction of all CNA-affected regions that are subclonal (Frac. CAN subclonal = 0.66), corresponding to the copy-number profiles shown in Figure 1 V-W. **Abbreviations:** CK8: cytokeratin 8; CNA: copy number alteration; Frac: Fraction; H&E: hematoxylin and eosin; UCS: uterine carcinosarcoma.


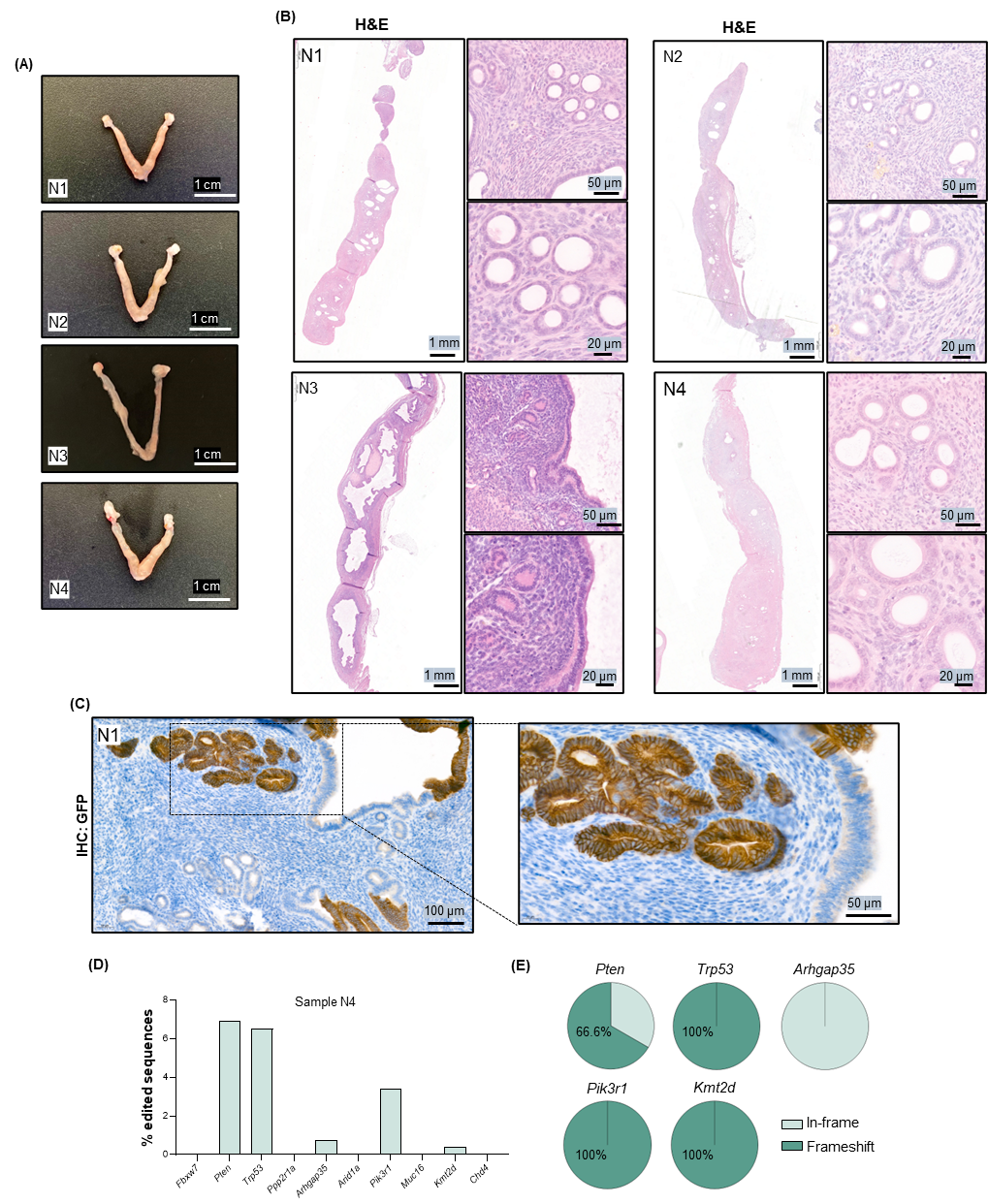


**Supplementary Figure S6. Histologically normal endometrium harbors mutations in targeted genes**. **(A)** Macroscopic images of electroporated uteri corresponding to normal endometrial histology of samples N1, N2, N3 and N4. **(B)** H&E staining corresponding to the normal endometria N1, N2, N3 and N4. **(C)** Representative images of GFP IHC analysis in normal-type endometrial sections. **(D)** Frequency of edited sequences in sample N4 (normal type lesion) is shown in percentage. **(E)** Pie charts showing the proportion of mutation types in edited genes from the normal type sample N4. **Abbreviations**: *Arhgap35*: rho GTPase activating protein 35; *Arid1a*: AT-rich interaction domain 1A; *Chd4*: chromodomain-helicase-DNA-binding protein 4; EP: electroporated; *Fbxw7*: F-box and WD repeat domain containing 7; GFP: green fluorescent protein; H&E: hematoxylin and eosin; IHC: immunohistochemistry; *Kmt2d*: lysine methyltransferase 2D; *Muc16*: mucin 16; N: normal; *Pik3r1*: phosphoinositide-3-kinase regulatory subunit; *Ppp2r1a*: protein phosphatase 2 scaffold subunit alpha; *Pten*: phosphatase and tensin homolog; RCA: rolling circle amplification; *Trp53*: transformation related protein 53; SEIC: serous endometrial intraepithelial carcinoma; TSG: tumor suppressor gene; UCS: uterine carcinosarcoma.

**Supplementary references**

1. Navaridas R, Vidal‐Sabanés M, Ruiz‐Mitjana A, Perramon‐Güell A, Megino‐Luque C, Llobet‐Navas D, et al. Transient and DNA‐free in vivo CRISPR/Cas9 genome editing for flexible modeling of endometrial carcinogenesis. Cancer Commun (Lond). 2023;43(5):620–4.

2. Eritja N, Llobet D, Domingo M, Santacana M, Yeramian A, Matias-Guiu X, et al. A Novel Three-Dimensional Culture System of Polarized Epithelial Cells to Study Endometrial Carcinogenesis. Am J Pathol. 2010;176(6):2722–31.

3. Clement K, Rees H, Canver MC, Gehrke JM, Farouni R, Hsu JY, et al. CRISPResso2 provides accurate and rapid genome editing sequence analysis. Nat Biotechnol. 2019;37(3):224–6.

4. Pinello L, Canver MC, Hoban MD, Orkin SH, Kohn DB, Bauer DE, et al. Analyzing CRISPR genome-editing experiments with CRISPResso. Nat Biotechnol. 2016;34(7):695–7.

5. Park J, Lim K, Kim JS, Bae S. Cas-analyzer: an online tool for assessing genome editing results using NGS data. Bioinformatics. 2017;33(2):286–8.

6. Stirling DR, Swain-Bowden MJ, Lucas AM, Carpenter AE, Cimini BA, Goodman A. CellProfiler 4: improvements in speed, utility and usability. BMC Bioinformatics. 2021;22(1):433.

7. R: The R Project for Statistical Computing [Internet]. [cited 2025 Aug 6]. Available from: https://www.r-project.org/

8. Wickham H, Averick M, Bryan J, Chang W, McGowan LD, François R, et al. Welcome to the Tidyverse. Journal of Open Source Software. 2019;4(43):1686.

9. Kolde R. pheatmap: Pretty Heatmaps [Internet]. 2025 [cited 2025 Aug 6]. Available from: https://cran.r-project.org/web/packages/pheatmap/index.html

10. Bankhead P, Loughrey MB, Fernández JA, Dombrowski Y, McArt DG, Dunne PD, et al. QuPath: Open source software for digital pathology image analysis. Sci Rep. 2017;7(1):16878.

11. Kassambara A. fastqcr: Quality Control of Sequencing Data [Internet]. 2023 [cited 2025 Aug 6]. Available from: https://cran.r-project.org/web/packages/fastqcr/index.html

12. Li H. Aligning sequence reads, clone sequences and assembly contigs with BWA-MEM [Internet]. arXiv; 2013 [cited 2025 Aug 6]. Available from: http://arxiv.org/abs/1303.3997

13. Van der Auwera GA, Carneiro MO, Hartl C, Poplin R, Del Angel G, Levy-Moonshine A, et al. From FastQ data to high confidence variant calls: the Genome Analysis Toolkit best practices pipeline. Curr Protoc Bioinformatics. 2013;43(1110):11.10.1-11.10.33.

14. shahcompbio/HMMcopy [Internet]. ShahCompBio; 2024 [cited 2025 Aug 6]. Available from: https://github.com/shahcompbio/HMMcopy

15. Adalsteinsson VA, Ha G, Freeman SS, Choudhury AD, Stover DG, Parsons HA, et al. Scalable whole-exome sequencing of cell-free DNA reveals high concordance with metastatic tumors. Nat Commun. 2017;8(1):1324.

16. AnnotationHub: Access the AnnotationHub Web Service [Internet]. [cited 2025 Aug 6]. Available from: https://bioconductor.org/packages/devel/bioc/vignettes/AnnotationHub/inst/doc/AnnotationHub.html

17. Rouatbi N, McGlynn T, Al-Jamal KT. Pre-clinical non-viral vectors exploited for in vivo CRISPR/Cas9 gene editing: an overview. Biomater Sci. 2022;10(13):3410–32.

18. de Bruijn I, Kundra R, Mastrogiacomo B, Tran TN, Sikina L, Mazor T, et al. Analysis and Visualization of Longitudinal Genomic and Clinical Data from the AACR Project GENIE Biopharma Collaborative in cBioPortal. Cancer Res. 2023;83(23):3861–7.

19. Gao J, Aksoy BA, Dogrusoz U, Dresdner G, Gross B, Sumer SO, et al. Integrative analysis of complex cancer genomics and clinical profiles using the cBioPortal. Sci Signal. 2013;6(269):pl1.

20. cBioPortal for Cancer Genomics [Internet]. [cited 2025 Jan 8]. Available from: https://www.cbioportal.org/study/summary?id=ucs_jhu_2014%2Cucs_tcga%2Cucec_cptac_2020%2Cucec_ancestry_cds_msk_2023%2Cucec_msk_2018%2Cucec_tcga%2Cucec_ccr_msk_2022%2Cucec_ccr_cfdna_msk_2022
